# A Novel Legumain-Like Protease in *Macrobrachium nipponense*: Identification, Characterization, and Function Analysis in Ovary Maturation

**DOI:** 10.3389/fendo.2022.858726

**Published:** 2022-03-24

**Authors:** Sufei Jiang, Yiwei Xiong, Wenyi Zhang, Junpeng Zhu, Dan Cheng, Yongsheng Gong, Yan Wu, Hui Qiao, Hongtuo Fu

**Affiliations:** ^1^ Key Laboratory of Freshwater Fisheries and Germplasm Resources Utilization, Ministry of Agriculture, Freshwater Fisheries Research Center, Chinese Academy of Fishery Sciences, Wuxi, China; ^2^ Wuxi Fisheries College, Nanjing Agricultural University, Wuxi, China

**Keywords:** *Macrobrachium nipponense*, *legumain-like* gene, ovary maturation, mRNA expression, RNA interference

## Abstract

*Legumain*, also called *aspartic endopeptidase* (AEP), is a member of the cysteine protease family and is involved in various physiological processes. In this study, we analyzed the characteristics of a novel *legumain-like* (named *Mn-Lel*) in the female oriental river prawn, *Macrobrachium nipponense*, which is involved in ovary maturation. The *Mn-Lel* is 1,454 bp in length, including a 1,290-bp open reading frame that encodes 430 amino acids. qPCR analysis indicated that *Mn-Lel* is specifically highly expressed in the hepatopancreas and ovaries of female prawns. It is rarely expressed in embryogenesis, weakly expressed in early larval development stages, and then significantly increased after metamorphosis, which indicated that *Mn-Lel* is not a maternal gene and mainly plays a role in adults. During the different ovarian stages, *Mn-Lel* expression in the hepatopancreas had no obvious rules, while its expression in the ovaries had a significant peak in stage III. *In situ* hybridization studies revealed that *Mn-Lel* is localized in the oocyte of the ovary. Changes in the gonadosomatic index confirmed the inhibitory effects of *Mn-Lel* dsRNA on ovary maturation. These results suggest that *Mn-Lel* has a key role in promoting ovary maturation.

## Introduction

Proteolysis in nature is mainly performed by proteases. Protease can be divided into several types according to the corresponding substrates, such as carboxyl peptidase and endopeptidase (1). Endopeptidase, which cleaves from the inside of the peptide chain, includes serine proteases, cysteine proteases, aspartic proteases, threonine proteases, and metalloproteases, and is characterized by its catalytic active central group (2, 3).

*Legumain*, also called aspartic endopeptidase, is a newly discovered member of the cysteine protease family, first identified in asparagus bean and asparagus bean plants. Like other family members, *legumain* is mainly located in the lysosome of cells and is involved in various physiological processes such as the degradation of basal egg whites (4). It can specifically hydrolyze the asparagine residue carboxy-terminal peptide bond. In recent years, significant progress has been made in the study of the structure and function of *legumain* genes and protein in plants, parasites, and mammals (5, 6). Studies in mammals have shown that it is involved in the pathophysiological process of immunity, anti-bone dissolution, and the occurrence and development of tumors (7, 8). The high expression level of *legumain* is related to tumor differentiation, tumor initiation and development, and tumor invasion (9–12). However, few studies have investigated *legumain* in normal growth development and reproduction.

*Macrobrachium nipponense*, also known as the oriental river prawn, is an economically important freshwater species and has been widely farmed not only in China but also in many Asian countries (13). Its production exceeded 240,000 tons in China in 2020 (14). The sexual maturity period of *M. nipponense* is short. In the breeding season, the ovary’s maturation of prawns is accelerated with the increase of water temperature. It only takes approximately 45 days from hatching to sexual maturity to laying eggs when the water temperature exceeds 28°C (15). Rapid mass self-reproduction in the pond produces a large number of offspring, which leads to a reduction in the resilience and survival rate and an increase in hypoxia risk. High-density farming also affects the market size of prawns and then seriously affects the economic benefits of prawn farming. It has been a problem of prawn farming for many years. To address this, transcriptomes of five ovary stages of *M. nipponense* were constructed to detect the key pathways and genes that play important roles in ovary maturation (16). The lysosomal pathway was detected to be the most significant enriched Kyoto Encyclopedia of Genes and Genomes (KEGG) pathway in the vitellogenesis stages. A novel *legumain-like* gene in this pathway showed remarkably expression variation, suggesting that it might have specific roles in *M. nipponense* ovary maturation.

Therefore, the present study aimed to characterize the structure of this *legumain-like* gene of *M. nipponense* and study its expression pattern and subcellular localization. Furthermore, we investigate its function in ovarian maturation. The results of this study were expected to provide a theoretical basis for revealing the regulation mechanism of ovarian development and solving the problem of fast sexual ripening in the *M. nipponense* aquaculture industry.

## Materials and Methods

### Sample Collection of *Macrobrachium nipponense* and Tissue Preparation

No endangered or protected species were involved in this study, and all experimental protocols and methods were approved in July 2021 (Authorization No. 20210715002) by the Animal Care and Use Ethics Committee in the Freshwater Fisheries Research Center (Wuxi, China).

Samples of female *M. nipponense* (n = 30, BW ± SD: 0.72 ± 0.19 g) used in gene cloning and expression analysis were obtained from Dapu scientific experimental base in Freshwater Fisheries Research Center (Wuxi, China). All prawns were adult and healthy, which were kept in recirculating glass aquarium system under the same environment for at least 1 week. Seven tissues (muscles, M; heart, H; ovary, O; cerebral ganglion, Cg; eyestalks, E; hepatopancreas, He; and gills, G) were collected from adult female prawns (n = 5). Ovarian stage division was according to a previous study (17). The ovaries of living prawn can be distinguished of different stages by color: OI (undeveloped stage, transparent), OII (developing stage, yellow), OIII (nearly-ripe stage, light green), OIV (ripe stage, dark green), and OV (spent stage, gray). Both hepatopancreas and ovaries from 5 stages were collected (n = 5).

The embryos and larvae were sampled according to the criteria of previous studies (17–19). The embryonic stages (n = 20) comprised cleavage stage (CS), blastula stage (BS), gastrulation stage (GS), nauplius stage (NS), and zoea stage (ZS). The larval stages (n = 10) were as follows: L1 (the 1st day after hatching), L5 (the 5th day after hatching), L10 (the 10th day after hatching), L15 (the 15th day after hatching), PL1 (the 1st day after metamorphosis), PL5 (the 5th day after metamorphosis), PL10 (the 10th day after metamorphosis), PL15 (the 15th day after metamorphosis), and PL25 (the 25th day after metamorphosis). All tissues were stored immediately in the liquid nitrogen for further study.

### RNA Isolation and cDNA Synthesis

Tissues weighing 0.1 g were used for total RNA isolation. RNAiso Plus Reagent (TaKaRa, Maebashi, Japan) was used for RNA extraction. Agarose electrophoresis (1.5% gel) was used to access RNA quality, and a NanoDrop 1000 was employed to measure the concentration of RNA. An RNA PCR Kit was used to synthesize cDNA (TaKaRa, Japan).

### *Legumain*-Like Sequence Cloning, Phylogenetic, and Structural Analyses

The full-length *legumain-like* cDNA sequence was obtained from the five ovary stages transcriptomes of *M. nipponense* (NCBI accession: SAMN11603268–SAMN11603282, Bioproject PRJNA541783). In order to verify the open reading frame sequence (ORF) of *legumain-like* gene, a pair of primers *Mn-Lel*-F and *Mn-Lel*-R was designed (listed in **Table 1**). PCR products were detected on 1.5% agarose gel and then sent to the company for sequencing (Sangon Biotech Shanghai Co., Shanghai, China).

**Table 1:** Primers used in this study.

Primer	Primer sequence (5′–3′)
*Mn-Lel* F (ORF)	AACCTGAGAATTGGAATCTTAG
*Mn-Lel* R (ORF)	GAGTTTTACATTTACTCGATGC
*Mn-Lel* F (qPCR)	GTTTTATGTAACGACCTCGGCTG
*Mn-Lel* R (qPCR)	GTGTGATTGGTAATTGCACTCGT
*EIF*-F (qPCR)	CATGGATGTACCTGTGGTGAAAC
*EIF*-R (qPCR)	CTGTCAGCAGAAGGTCCTCATTA
*Mn-Lel* probe	ACAGCCGAACTGGTCGCCAATGTACGTGTCTC
*Mn-Lel* anti-probe	GAGACACGTACATTGGCGACCAGTTCGGCTGT
*Mn-Lel* iF (RNAi)	GATCACTAATACGACTCACTATAGGGTGGAGGATCTCATTCCAAGG
*Mn-Lel* iR (RNAi)	GATCACTAATACGACTCACTATAGGGTATCGGCTCCGAAGTTGTTC
*GFP* iF (RNAi)	GATCACTAATACGACTCACTATAGGGTCCTGGTCGAGCTGGACGG
*GFP* iR (RNAi)	GATCACTAATACGACTCACTATAGGGCGCTTCTCGTTGGGGTCTTTG

The *legumain-like* gene sequence was analyzed using BLAST programs (http://www.ncbi.nlm.nih.gov/BLAST/), and SignalP4.1 was applied in signal peptide predicting (http://www.cbs.dtu.dk/services/SignalP-3.0/). The *legumain-like* amino acid sequences were used to generate the phylogenetic trees with MEGA5.0 based on the neighbor-joining (NJ) method, and the bootstrapping replications were 1,000 (20, 21). Sequence alignment was built with DNAMAN 6.0 software (18). The known sequences of other animals were obtained from the NCBI database (http://www.ncbi.nlm.nih.gov/). NetNGlyc-1.0 (https://services.healthtech.dtu.dk/service.php?NetNGlyc-1.0) was employed to predict potential sites of N-linked glycosylation.

### Quantitative Real-Time PCR Analysis

Quantitative Real-Time PCR (qPCR) was used to quantify spatiotemporal mRNA expression levels of the *legumain-like* gene. *EIF* gene (*eukaryotic translation initiation factor 5A*) was used as the internal reference for both tissue distribution and development stage expression analysis (listed in **Table 1**) (22). The qPCR system was detailed in a previous study (23). The expression levels were calculated based on the 2^− ΔΔCT^ method (24). SPSS 23.0 software was used to do statistical analyses, and one-way ANOVA and two-tailed t-test were used to analyze statistical differences. All data were confirmed to the homogeneity of variance and normal distribution. All qPCR data were described as mean ± SD, and a significant difference was indicated by *p* < 0.05.

### *In Situ* Hybridization

The ovaries were dissected from mature female prawns and fixed in 4% paraformaldehyde in phosphate-buffered saline (PBS; pH 7.4) at 4°C overnight. *In situ* hybridization (ISH) study was performed on 4-μm-thick formalin-fixed paraffin-embedded sections using Zytofast PLUS CISH implementation kit (ZytoVision GmBH, Bremerhaven, Germany) (25). The blank control groups were routine H&E staining sections without probe poured, and the negative control groups had antisense probes poured. The mRNA locations of Mn-Lel were analyzed with sense probe poured over slides. The anti-sense and sense probes in this study with digoxin signal were designed by Primer5 software based on the cDNA sequence of Mn-Lel and synthesized by Shanghai Sangon Biotech Company (Shanghai, China) (**Table 1**). All slides were examined under a light microscope for evaluation.

### RNA Interference

The RNA interference (RNAi) primers of *legumain-like* and green fluorescent protein (*GFP*) gene, which contained the T7 promoter, were designed for using Snap Dragon (http://www.flyrnai.org/cgibin/RNAifind_primers.pl) (listed in **Table 1**). The *GFP* gene was selected as a control (26). Transcript AidTMT7 High Yield Transcription kit (Fermentas, Inc., USA) was used to synthesize *in vitro* dsRNA of both *legumain-like* and *GFP*.

In order to calculate the change degree of gonadosomatic index (GSI) better and more clearly, the ovary development level of experimental prawns should be relatively consistent at the beginning of the experiment. At the beginning of the RNAi experiment, most females were in the ovary stage IV. Therefore, stage IV prawns were selected to ensure a sufficient number of experimental prawns. One hundred healthy female prawns (0.83 ± 0.11 g) in stage IV were randomly divided into an experimental group and a control group (N = 50). A 17-day RNAi experiment was carried out (water temperature 25°C ± 1°C). Both experimental and control groups were injected with 4 μg/g.b.w of ds-*legumain-like* and ds-*GFP* each, and the injection site is pericardial cavity membrane (27). Ds-RNA was injected every 5 days, and five prawns in each group were randomly sampled on the 1st, 9th, and 17th days. By silencing the *legumain-like* gene, interference efficiency in both hepatopancreas and ovary and the GSI (gonadal weight/body weight × 100%) were calculated. Ten prawns were randomly sampled from both groups on the 1st, 9th, and 17th days, and the body and ovary weight were recorded.

## Results

### Characterization and Structural Analysis of *Mn-Lel*

**Figure 1:**
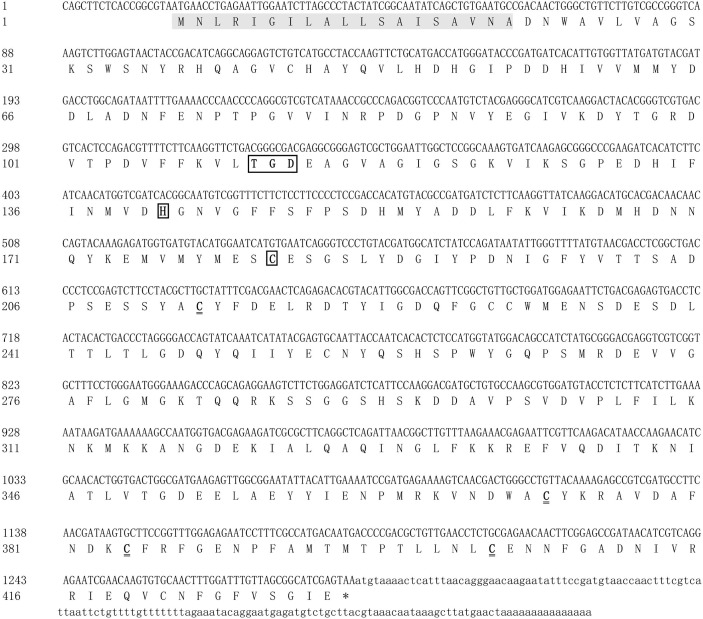
Nucleotide and deduced amino acid sequences of *Mn-Lel* in *Macrobrachium nipponense*. The shaded area indicates the signal peptide. The black underlined boxes represent the conserved TGD motif (Thr^110^-Gly^111^-Asp^112^) and His^140^ and Cys^181^ catalytic dyad in the C13 superfamily domain. The double underline represents highly conserved cysteine residues were located in the 212th, 371th, 383th, 403rd, and 420th. The asterisk (*) represents the termination codon.

The full-length *legumain-like* cDNA sequence of *M. nipponense* was 1,454 bp, named as *Mn-Lel* (GenBank No. OM317599), including 19 and 145 bp of 5′ and 3′ untranslated region (UTR), respectively. It had a polyadenylation signal (AATAAA) and a poly(A) tail in the 3′-UTR, indicating the integrity of the *Mn-Lel* gene (**Figure 1**). The ORF of *Mn-Lel* was 1,290 bp and encoded 430 amino acids, and the estimated molecular mass was 48.05 kDa; the theoretical pI was 4.44. SignalP software analysis showed that *Mn-Lel* had a signal peptide with a length of 19 amino acid residues, and the predicted tangent point was between Ala^19^ and Asp^20^. The results of BLASTP showed that the 21st to 278th amino acid residues constituted a C13 superfamily domain of peptidase. There was a conserved TGD motif (Thr^110^-Gly^111^-Asp^112^) and His^140^ and Cys^181^ catalytic dyad in the C13 superfamily domain. Several highly conserved cysteine residues were located in the 212th, 371th, 383th, 403rd, and 420th. We found that there were no potential N-linked glycosylation sites in *Mn-Lel* based on NetNGlyc-1.0.

### Phylogenetic and Sequence Alignment Analysis of *Mn-Lel*

**Figure 2:**
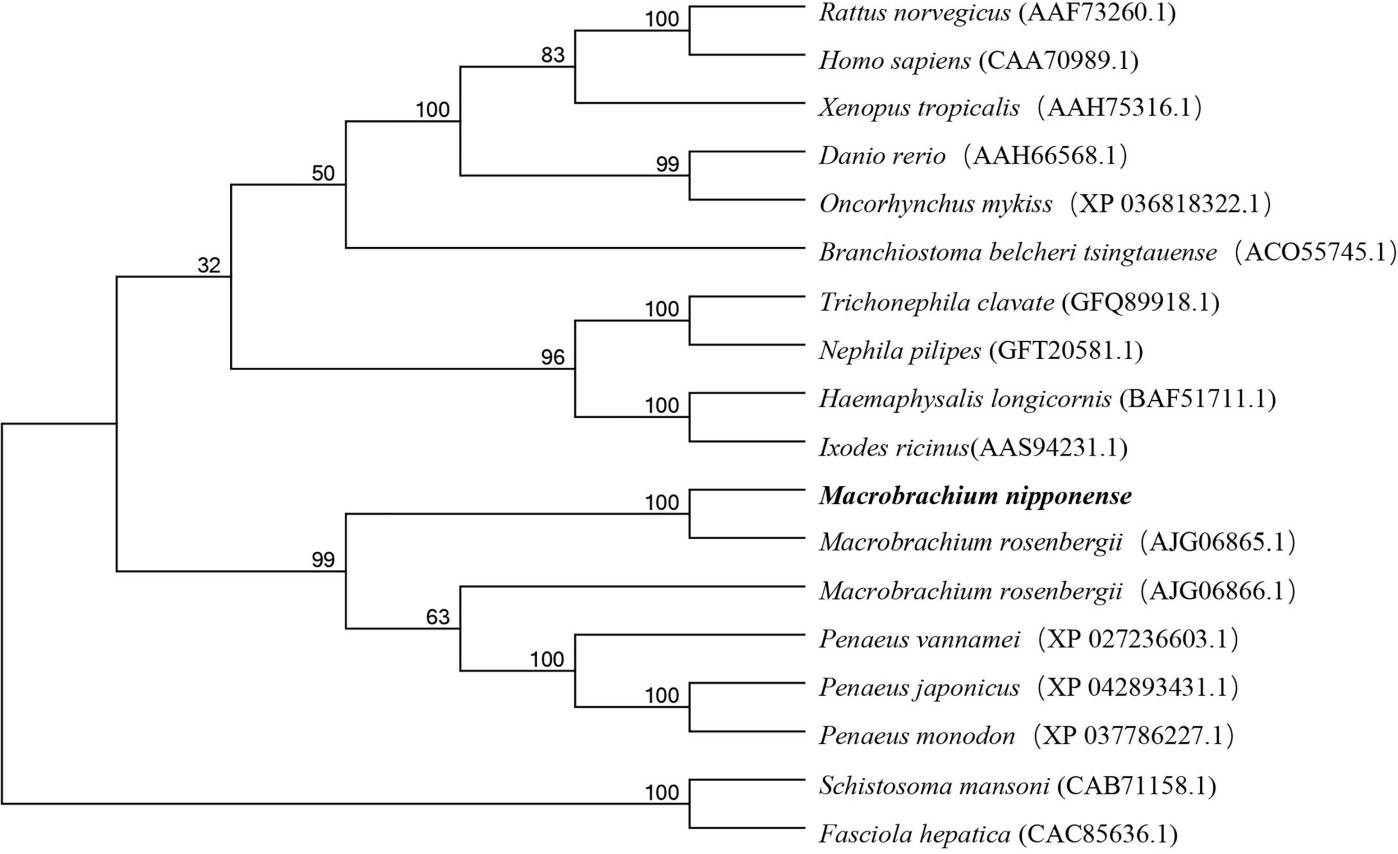
Phylogenetic tree of *Mn-Lel* amino acid sequence in different groups. The numbers below the node indicate the bootstrap value.

Several amino acid sequences of *legumain* and *legumain-like* in vertebrates and invertebrates were deduced, and a phylogenetic tree was built to elucidate the phylogenetic relationships based on the NJ method (**Figure 2**). The results showed that *legumain* and *legumain-like* were highly conserved in evolution; all *legumain* and *legumain-like* could be divided into two branches. The *Mn-Lel* and *legumain-like* protein of *Macrobrachium rosenbergii* (AJG06865.1) were isolated and clustered into one branch with other crustaceans. The crustacean *legumains* were then grouped with vertebrates and insects. The *legumain* from the parasite was isolated to be one branch.

**Figure 3:**
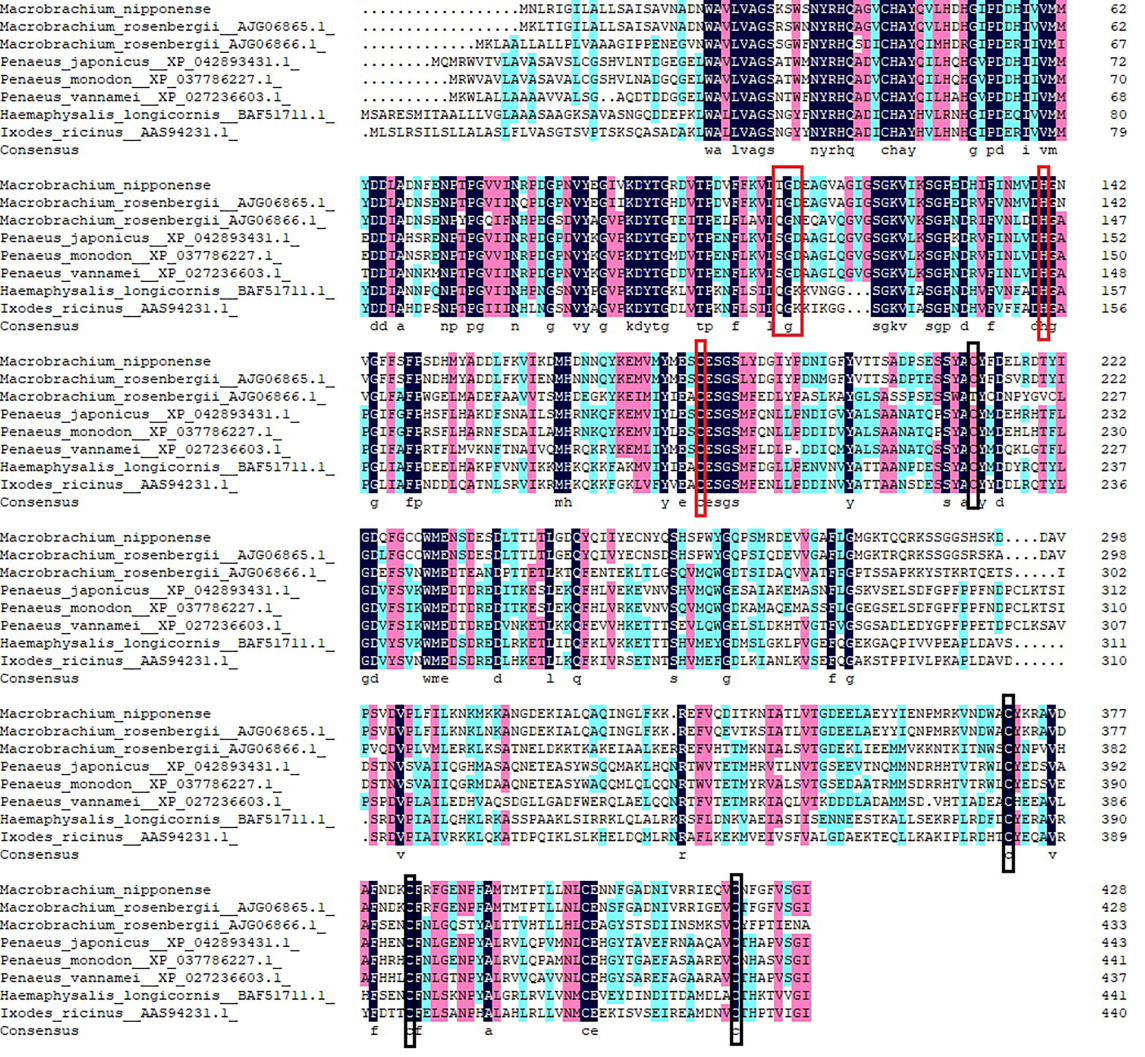
Multiple amino acid alignment and structure prediction of *legumains*. TGD motif and His-Cys Catalytic dyad are represented by red boxes. Highly conserved cysteine residues are represented by black boxes. *Legumain-like* protein (*Macrobrachium rosenbergii*, AJG06865.1), *legumain-like* protein (*M. rosenbergii*, AJG06866.1), *legumain-like* (*Penaeus japonicus* XP_042893431.1), *legumain-like* (*Penaeus monodon*, XP_037786227.1), *legumain-like* (*Penaeus vannamei*, XP_027236603.1), *legumain* (*Haemaphysalis longicornis* BAF51711.1), and *legumain-like* (*Ixodes ricinus* AAS94231.1).

Multiple sequence alignment was performed using DNAMAN 6.0 to compare *Mn-Lel* gene with *legumain* and *legumain-like* of crustaceans and insects in Arthropoda, including *M. rosenbergii*, *Penaeus japonicus*, *Penaeus monodon*, *Penaeus vannamei*, *Haemaphysalis longicornis*, and *Ixodes ricinus*. The homology with these species was 86.06%, 39.60%, 40.71%, 41.15%, 41.81%, 39.96%, and 37.25%, respectively (**Figure 3**).

### Spatiotemporal Expression Patterns of *Mn-Lel*

**Figure 4:**
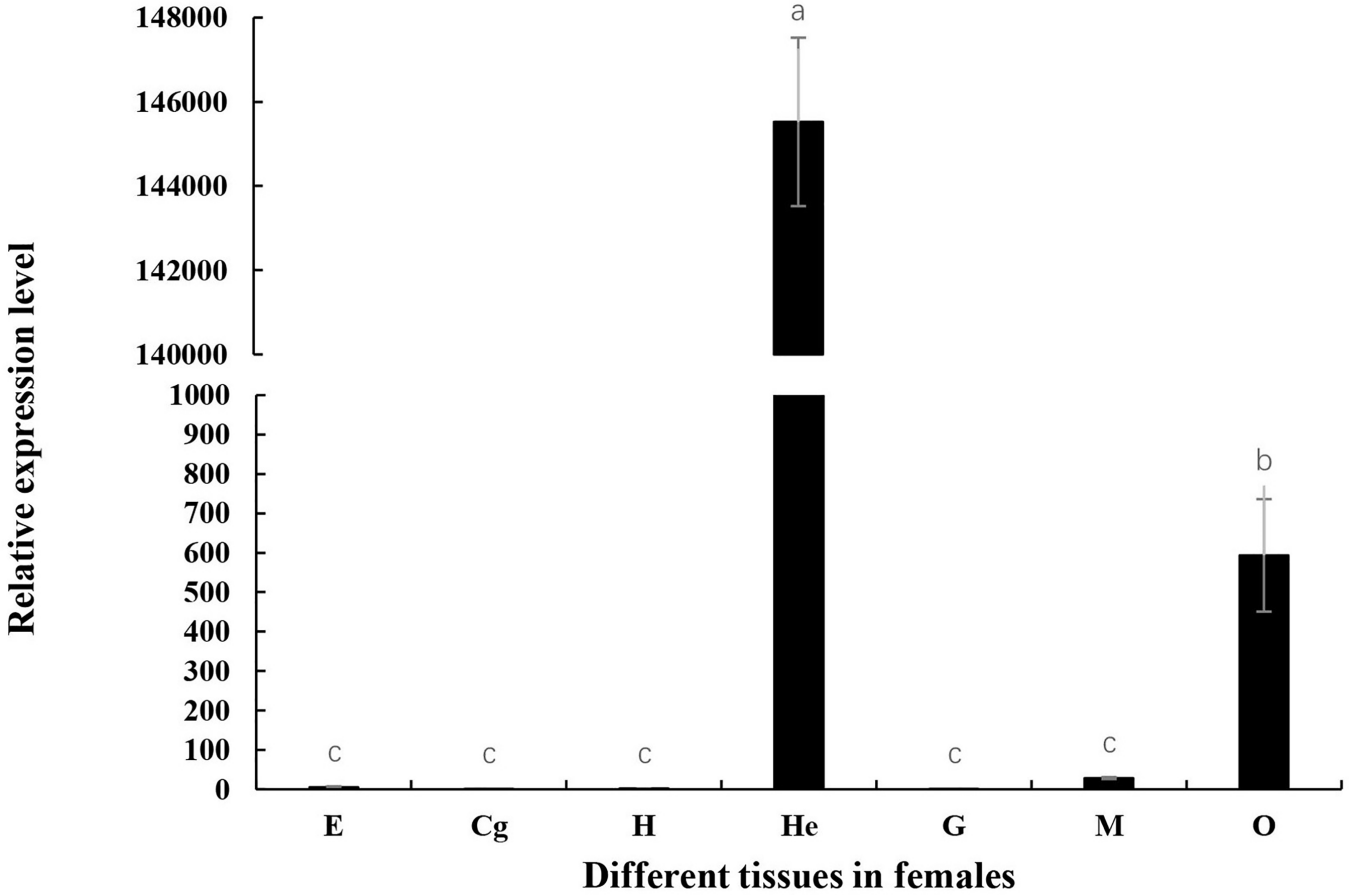
Tissue distribution of *Mn-Lel*. E, eyestalk; Cg, cerebral ganglion; H, heart; He, hepatopancreas; G, gill; M, muscle; O, ovary. Data are shown as mean ± SD (n = 5). Different letters denote significant differences (*p* < 0.05). Bars with different letters were considered significant at *p* < 0.05.

Tissue expression of *Mn-Lel* was monitored by qPCR using cDNA templates originating from the female muscles (M), heart (H), ovary (O), cerebral ganglion (Cg), eyestalks (E), hepatopancreas (He), and gills (G) tissues as displayed in **Figure 4**. The Ct value of qPCR revealed that *Mn-Lel* was hardly expressed in eyestalks, cerebral ganglion, heart, and gills but was weakly expressed in the muscle. High expression levels were detected in the hepatopancreas and ovary. *Mn-Lel* was highly expressed in both these tissues, but the expression level in the hepatopancreas was nearly 250 times more than that in the ovaries (*p* < 0.01).

**Figure 5:**
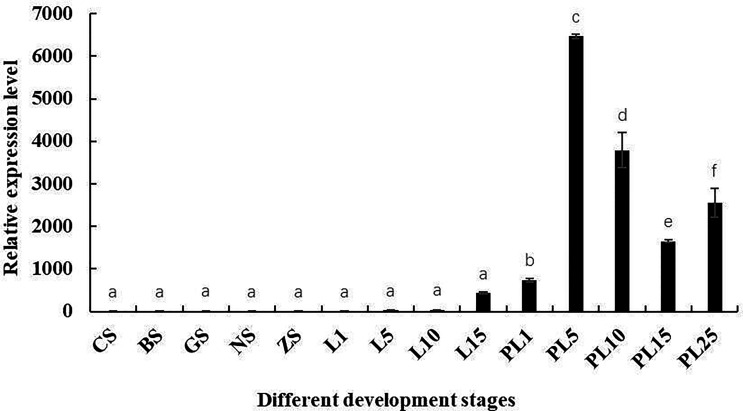
The expression pattern of the *Mn-Lel* in developmental stages of *Macrobrachium nipponense*. CS, cleavage-stage; BS, blastocyst stage; GS, gastrulation stage; NS, nauplius stage; ZS, zoea stage; L1, the 1st-day larvae after hatching; L5, the 5th-day larvae after hatching; L10, the 10th-day larvae after hatching; L15, the 15th-day larvae after hatching; PL1, post-larval stage of the 1st day; PL5, post-larval stage of the 5th day; PL10, post-larval stage of the 10th day; PL15, post-larval stage of the 15th day; PL-25M, post-larval stage of the 25th day. Data are presented as the mean ± SD (n = 6). *p* < 0.05 was considered to be statistically significant. Bars with different letters were considered significant at p < 0.05.

*Mn-Lel* expression during embryo and larva development was also investigated (**Figure 5**). According to the Ct value of qPCR, *Mn-Lel* was hardly expressed in the embryo development stages (from CS stage to ZS stage), and it was also hardly expressed until the 15th day after hatching (L15) (*p* < 0.05). *Mn-Lel* expressed significantly from L5 to the 1st day after metamorphosis (PL1) and then dramatically increased PL5 by about 10 times than PL1 (*p* < 0.05), which reached the top. In later larval development stages (from PL5 stage to PL25 stage), the *Mn-Lel* showed a significant fluctuation trend (*p* < 0.05).

**Figure 6:**
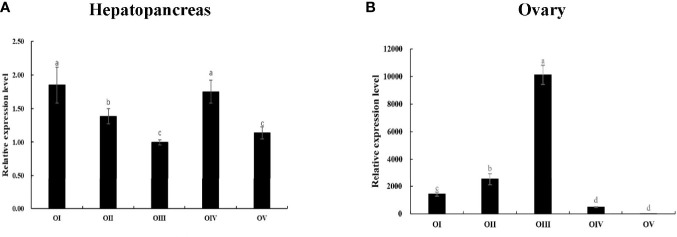
Expression patterns of *Mn-Lel* in the hepatopancreas **(A)** and ovary **(B)** at different ovarian stages. Different ovarian stages expressions: OI, undeveloped stage; OII, developing stage; OIII, nearly-ripe stage; OIV, ripe stage; OV, spent stage. Statistical analyses were performed with one-way ANOVA. Data are shown as mean ± SD (n = 5). Different letters denote significant differences (*p* < 0.05). Bars with different letters were considered significant at *p* < 0.05.

Subsequently, *Mn-Lel* is expressed in the hepatopancreas and ovaries during the ovary maturation cycle. According to the Ct values, *Mn-Lel* was highly expressed during the ovary maturation cycle that was examined. The Ct value of *Mn-Lel* gene in hepatopancreas maintained a remarkably high level in all five ovary stages (**Figure 6A**). It was found that *Mn-Lel* was higher in stages I and IV (*p* < 0.05), and its expression was slightly lower as detected in the other three stages (*p* < 0.05). In ovaries (**Figure 6B**), the Ct value of *Mn-Lel* gene also showed a high expression level. The *Mn-Lel* expression level doubled obviously from stage I to II (*p* < 0.05). Then its expression significantly increased from stage II to III approximately five-fold (*p* < 0.05) and reached the peak in stage III. Subsequently, it decreased dramatically from stage III to IV (*p* < 0.05).

### *Mn-Lel* Localization in Five Ovary Stages by *In Situ* Hybridization

**Figure 7:**
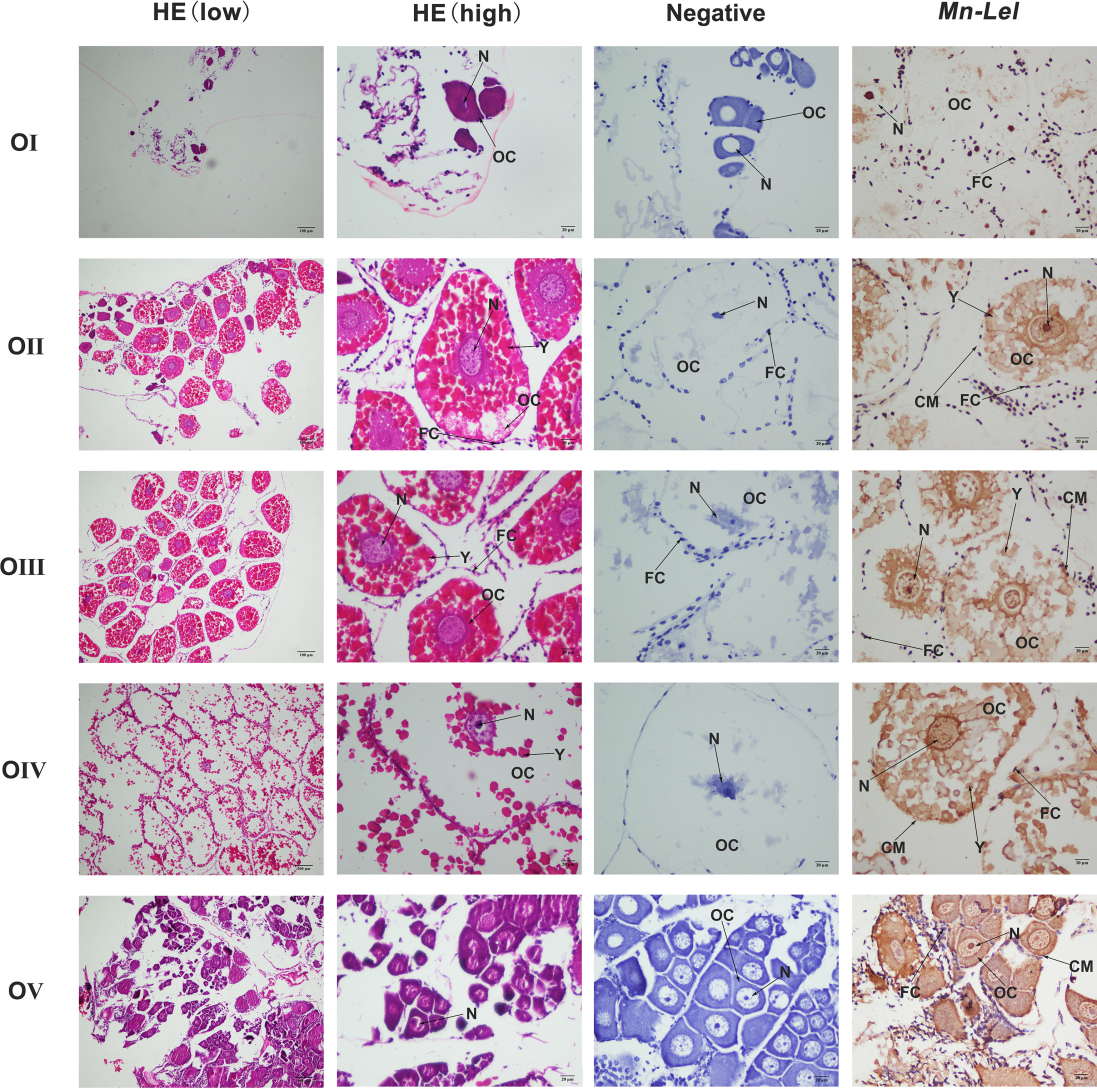
Location of *Mn-Lel* detected in the ovary by *in situ* hybridization. A photograph of *Macrobrachium nipponense* ovary in ovarian cycle. HE, H&E staining paraffin section; OC, oocyte; N, nucleus; CM, cytoplasmic membrane; Y, yolk granule; FC, follicle cell; FM, follicle membrane. Scale bars, ×100 (low), ×400 (high).

Periodic changes of ovarian development were clearly shown in the paraffin section of **Figure 7**. In stage I, the space of the ovarian cavity is large, the nucleus of the oocyte is round and large, and the protoplasm is less and transparent. In stage II, the ovarian cavity began to shrink significantly, the oocyte was closely arranged, and the nucleus is relatively small. In stage III, the ovarian cavity becomes narrow, and a layer of vacuoles appears around the oocyte. The oocyte begins to deposit yolk. In stage IV, oocyte development was completed, and the cytoplasm was filled with a large number of oocytes. In stage V, the ovary was filled with empty follicles and a few stage I oocytes. ISH was used to detect the *Mn-Lel* localization in ovary stages (**Figure 7**). The results indicated that the *Mn-Lel* signal was detected in all five stages. The *Mn-Lel* signal could be found in oocytes including the nucleus, follicle cell, yolk granule, cytoplasmic membrane, and follicle membrane, while follicle membrane, cytoplasmic membrane, and nucleus were the main expressed sites. The color intensity of the signal showed that *Mn-Lel* is mainly expressed in the membrane of the follicle, cytoplasmic membrane, and nucleus. It showed that the signal of *Mn-Lel* in the ovary was significantly enhanced during stage II (primary vitellogenesis) and stage III (secondary vitellogenesis) and gradually weakened in other stages.

### Effect of Knocking Down *Mn-Lel* on Ovary Maturation of *Macrobrachium nipponense*

**Figure 8:**
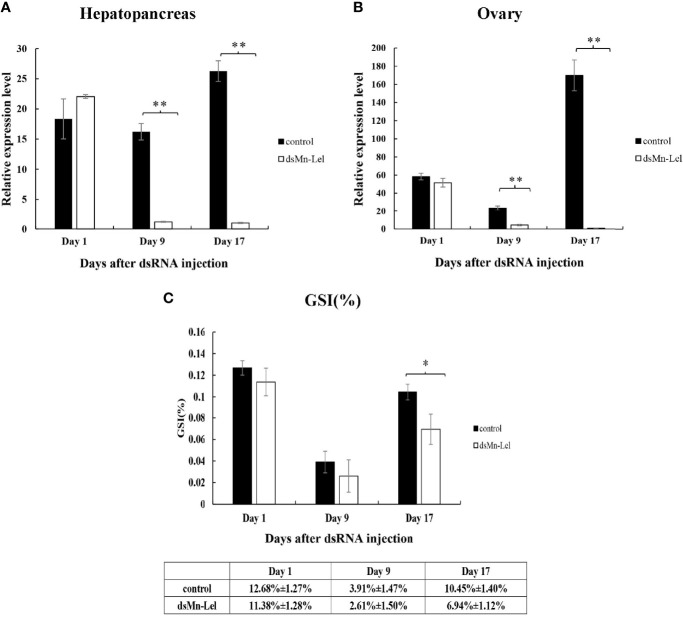
Function analysis of *Mn-Lel* by RNAi. **(A)** Efficiency of RNAi-*Mn-Lel* knockdown in hepatopancreas. **(B)** Efficiency of RNAi-*Mn-Lel* knockdown in ovary. **(C)** Changes in gonadosomatic index (GSI) (%) of female *Macrobrachium nipponense* after injection with *Mn-Lel* dsRNA. Data are presented as mean ± SD (n = 6); * denotes statistical significance of *p* < 0.05. ** denotes statistical significance of p < 0.01.

*Mn-Lel* was knocked down by a 3-week RNAi. The ovaries of both control and experimental groups were in stage IV (ripe stage, dark green) at the beginning, and as the experiment progressed, the ovaries emptied and became full again. The RNAi efficiency of *ds-Mn-Lel* in hepatopancreas and ovary was tested. In hepatopancreas, the *Mn-Lel* expression level was significantly downregulated by 92.67% on the 9th day after injection and then by 96.19% on the 17th day after injection (*p* < 0.01) (**Figure 8A**). In the ovary, the *ds-Mn-Lel* showed an obviously decreased efficiency by 81.49% on the 9th day and 99.41% on the 17th day after injection (*p* < 0.01) (**Figure 8B**). The GSI in both groups was also tracked and calculated (**Figure 8C**). In the beginning, the average GSI of control and experiment groups was 12.68% and 12.00%, respectively; however, the experiment progressed, and there were no obvious changes between the two groups (control: 3.91%, *ds-Mn-Lel*: 4.01%) on the 9th day after injection (*p* > 0.05). On the 17th day, the average GSI of the control group increased to 10.45%, while it was only 6.94% in the experiment group (*p* < 0.05).

## Discussion

*Legumain* has been previously reported in parasites and mammals. The precursor enzyme of *legumain* was relatively conserved and composed of signal-peptide, propeptide, and catalytic domain containing the active center of mature protease. *Legumain* of *Schistosoma mansoni* consisted of 429 amino acids (28). *H. longicornis* has two *legumain*s with a length of 442 amino acids (29, 30). Human and mouse *legumain* encoded 433 and 435 amino acid polypeptide chains, respectively (5). In this study, *Mn-Lel* encoded 430 amino acids, with structural characteristics similar to those of humans, arthropods, and schistosoma (5, 28, 31). The sequence included a signal peptide, a C13 superfamily domain of peptidase, containing a His-Cys catalytic dyad, which is necessary for *legumain* to exert catalytic activity, and was also a required structure for every C13 family member. There was also a conserved TGD motif (Thr^110^-Gly^111^-ASP^112^) in the C13 domain of *Mn-Lel*, which corresponded to mammalian RGD (Arg-Gly-Asp), *Schistosoma japonicum* KGD (Lys-Gly-Asp), and tick QGK (Gin-Gly-Lys) (5, 28, 31, 32). It was speculated that the mammalian RGD motif interacts with a component on the cell membrane to help *legumain* survive in the acidic environment of the lysosome. The largest difference here is that we did not find potential N-linked glycosylation sites in *Mn-Lel*, which suggested that the role of *Mn-Lel* might differ from that of conventionally reported *legumains*. Among vertebrates, the *legumain* protein sequence of zebrafish has more than 60% homology with *legumain* in some species; in particular, it has 66.7% homology with *legumain* in humans, indicating that *legumain* is highly conserved and plays an important role in evolution (9, 32, 33). Sequence alignment analysis results in the present study showed that the amino acid sequence homology of *Mn-Lel*, as well as *legumain-like* protein of *M. rosenbergii* (AJG06865.1) with other crustacean *legumains*, was not very high (about 40%). This indicated that these two crustacean *legumain-like* proteins may play a very important role with others in evolution.

The *legumains* expression has been studied most extensively in human tumors. In the examination of the normal tissues and a variety of tumor tissue, *legumain* had a low expression in normal tissue; the high expression was detected in many solid tumors, including breast, colon, lung, prostate, and ovarian tumors and malignant tumors of the central nervous system (34–37). However, there were relatively few studies on *legumain* expression in normal growth and tissues. *legumain* in schistosoma, nematodes, ticks, and other parasites is mainly expressed in the intestine and is responsible for the health of host hemoglobin solution (28, 30, 31, 38). *Legumain* in *Amphioxus Branchiostoma belcheri* was not widely distributed, and hepatic cecum and hind-gut were the only expression sites (39). In the present, the spatiotemporal expression pattern of *Mn-Lel* was analyzed by qPCR, and we found that *Mn-Lel* showed a significant difference in spatiotemporal expression and tissue specificity. In adult prawn tissues, *Mn-Lel* was specifically highly expressed in hepatopancreas and ovaries but weakly expressed or not expressed in other detected tissues, which were clearly different from immune-related *legumains* found in other species mentioned above. This suggested that the *Mn-Lel* might perform a different function. In zebrafish embryo development, *legumain* was universally expressed and was specifically expressed in hematopoietic tissues 22 h after fertilization, which was consistent with the universal expression profiles in mouse and human hematopoietic tissues (6, 33). *Mn-Lel* was rarely expressed in embryogenesis and weakly expressed in the early larval development of *M. nipponense*. It expressed significantly after metamorphosis, which indicated that *Mn-Lel* was not a maternal gene and mainly plays a role in adults.

To date, no *legumain* reports have been related to reproduction. In view of the high specific expression of *Mn-Lel* in the hepatopancreas and ovaries of *M. nipponense*, we subsequently studied its expression patterns in hepatopancreas and ovaries at five different ovary stages. *Mn-Lel*, as well as the other two *cathepsin L* genes discovered in transcriptomes of 5 ovary stages of *M. nipponense*, were significantly enriched in the lysosomal pathway in vitellogenesis stages (stage II to stage III) (16). Studies in crustaceans showed that vitellogenin was transported from the hepatopancreas to the oocyte through the hemolymph (40, 41). Ovary stage III was a rapid period of growth and proliferation of oocytes, which provided nutrients and energy. Previous studies (42, 43) suggested that two *cathepsin L* genes are mainly expressed in the hepatopancreas, and their expression levels in the ovary reached the maximum in stage III, which was similar to *Mn-Lel.* ISH results also showed that a clear *Mn-Lel* signal was detected in all five stages. The *Mn-Lel* signal could be found in oocytes including the nucleus, follicle cell, yolk granule, cytoplasmic membrane, and follicle membrane. It was well distributed in the nucleus and around the yolk. These results gave evidence that *Mn-Lel* plays a special role in ovary maturation like other *cathepsin L* genes. *Legumain*, as a protease, not only specifically hydrolyzes peptide bonds of asparagine residues. In addition to protein degradation, it could also hydrolyze and activate some precursor proteins (proenzyme and prohormone), or activate other proteolytic enzyme systems to participate in various physiological activities of the body (6). In parasites, studies on *legumain* are mainly confined to schistosoma, nematodes, and ticks. They were mainly involved in host hemoglobin degradation (28, 30, 38). Mammalian *legumains* are involved in many pathophysiological processes such as immunity, bone dissolution, and tumorigenesis (44–46). In addition, few functional studies on *legumain* have been conducted. In the present study, RNAi was used to illustrate the special roles of *Mn-Lel* in the ovary maturation of *M. nipponense*. The *ds-Mn-Lel* showed extreme inhibitory effects on *Mn-Lel* expression. GSI result intuitively showed the inhibitory effect on ovary maturation. The results demonstrate that *Mn-Lel* played an important role in yolk accumulation. Inhibiting the expression of *Mn-Lel* can effectively inhibit ovarian maturation.

## Conclusion

In the present study, a novel function *legumain-like* protease from ovary transcriptomes of *M. nipponense* was characterized and named as *Mn-Lel*. It was the first report about a reproduction that involved *legumain* gene in crustaceans. The expression, distribution, and function of *Mn-Lel* gene in *M. nipponense* were systematically analyzed by qRT-PCR, ISH, and RNAi. Our research results strongly prove that *Mn-Lel* plays a pivotal role in the ovarian maturation process of *M. nipponense*. This study enriched the molecular mechanisms of ovary maturation during the reproduction period of female *M. nipponense* and provided new insights for reproductive regulation mechanisms in crustaceans.

## Data Availability Statement

The original contributions presented in the study are included in the article/supplementary materials. Further inquiries can be directed to the corresponding authors.

## Ethics Statement

The animal study was reviewed and approved by Animal Care and Use Ethics Committee in the Freshwater Fisheries Research Center (Wuxi, China).

## Author Contributions

Designed the study: HQ and HF. Carried out the experiments and wrote the original draft: SJ and YX. Provided technical assistance: WZ and JZ. Methodology and data curation: SJ and YX. Resources: DC and YG. Software: YW. All authors have read and agreed to the published version of the manuscript.

## Conflict of Interest

The authors declare that the research was conducted in the absence of any commercial or financial relationships that could be construed as a potential conflict of interest.

## Publisher’s Note

All claims expressed in this article are solely those of the authors and do not necessarily represent those of their affiliated organizations, or those of the publisher, the editors and the reviewers. Any product that may be evaluated in this article, or claim that may be made by its manufacturer, is not guaranteed or endorsed by the publisher.
